# Stevens Johnson Syndrome and Toxic Epidermal Necrolysis: Maternal and Foetal Outcomes in Twenty-Two Consecutive Pregnant HIV Infected Women

**DOI:** 10.1371/journal.pone.0135501

**Published:** 2015-08-12

**Authors:** Lauren Knight, Gail Todd, Rudzani Muloiwa, Mushi Matjila, Rannakoe J. Lehloenya

**Affiliations:** 1 Division of Dermatology, Department of Medicine, University of Cape Town, Cape Town, South Africa; 2 Department of Paediatrics and Child Health, University of Cape Town, Cape Town, South Africa; 3 Department of Obstetrics and Gynaecology, University of Cape Town, Cape Town, South Africa; Centers for Disease Control and Prevention, UNITED STATES

## Abstract

**Introduction:**

Stevens-Johnson syndrome (SJS) and toxic epidermal necrolysis (TEN) form a spectrum of a rare and life-threatening cutaneous drug reaction. SJS/TEN in pregnancy poses largely unknown risk factors and outcomes for both the mother and foetus compared to the general population.

**Methods:**

We conducted a study of consecutive pregnant women admitted to single tertiary referral centre in South Africa with SJS/TEN over a 3 year period. They were all managed by the same medical team using the same protocols. We evaluated their underlying illnesses, offending drugs and the course of pregnancy and outcomes to determine factors influencing maternal and foetal outcomes.

**Results:**

We identified twenty-two women who developed SJS/TEN while pregnant, all of them HIV-infected. Their median age was 29 years. The majority 16/22 (73%) had SJS, the milder variant of the disease affecting < 10% body surface area. Nevirapine was the offending drug in 21/22 (95%) cases. All 22 of the mothers survived with 3/22 (14%) developing postpartum sepsis. Pregnancy outcomes were known in 18/22 women and 9/18 (50%) babies were delivered by caesarean section. There were 2 foetal deaths at 21 and 31 weeks respectively and both were associated with post-partum sepsis. Postnatal complications occurred in 5 cases, 3 involving the respiratory system and the other two being low birth weight deliveries. Eight placentae and one foetus were sent for histology and none showed macroscopic or microscopic features of SJS/TEN. On follow-up, only 12/20 children were tested for HIV at 6 weeks post-delivery and none of them were HIV-infected. All had received prophylactic ARVs including nevirapine.

**Conclusions:**

TEN, the severe form of the disease, was associated with poorer foetal outcomes. SJS/TEN-associated mortality is not increased in HIV-infected pregnant women. Maternal SJS/TEN does not seem to commonly manifest in the foetus.

## Introduction

Stevens-Johnson syndrome (SJS) and toxic epidermal necrolysis (TEN) are rare life-threatening cutaneous drug reactions, with skin and mucous membrane involvement that are considered a spectrum of the same disease. In SJS, there is <10% of epidermal detachment and in TEN there is >30% with SJS/TEN overlap lying between these two extremes.[1] The incidence of SJS/TEN is approximately 0,05–2 cases/million per year for the general population, in developed countries where the data is available. In patients with AIDS the incidence is estimated to be 1 to 2 per 1000 individuals.[2] The disease is associated with significant morbidity and mortality. In larger published series of patients, mortality has been reported to be between 4 and 60%, while long-term sequelae is as high as 50% in children and more than 80% in adults.[3–7][8–10]

Various risk factors for the development of SJS/TEN have been reported, including HIV infection, some HLA alleles, collagen vascular disease and age.[2,5,11] Sekula and colleagues conducted a survival analysis of a cohort of patients with SJS/TEN which validated the parameters of SCORTEN, a prognostic score for in-hospital mortality.[12] The score is based on seven risk indicators, these being: (1) age more than 40 years, (2) recent or current malignancy, (3) tachycardia more than 120 per minute, (4) blistering more than 10% of total affected body surface area, (5) serum urea more than 10 mmol/L, (6) bicarbonate less than 20 mmol/L, and (7) serum glucose more than 250 mg/dL.[13] In addition, the authors found that a recent infection was an independent predictor of mortality.[12] None of the patients evaluated in the survival analysis was reported to be pregnant. There is paucity of robust data relating to predictors of maternal and foetal outcomes in pregnant women who develop SJS/TEN. Almost all of the reported cases are single cases.[14]^-^

Pregnant women who develop SJS/TEN are a unique subset, possibly with different risk factors and outcomes for both the mother and the foetus compared to the general population. SJS/TEN can simultaneously affect the mother and the foetus and in the literature there are at least 5 such reported cases[15]. In pregnancy, low maternal body weight, high nevirapine (NVP) plasma levels and CD4 counts of greater than 250 cells/μl have been associated with an increase in the incidence of SJS/TEN[16]. Dube et al, in a matched case control study found that pregnancy itself increased the risk of developing SJS by 14 fold when HIV-infected women used NVP based regimens in pregnancy (OR14.28, p = 0.006, 95% CI 1.54–131.82).[16] In reviewed literature, a high survival rate is suggested for both the mother and fetus. Based on the current limited data, it seems mortality in pregnancy-associated SJS/TEN is lower than the general population. In pregnancy, the most significant effect of SJS/TEN on the unborn foetus has been shown to be an increased risk of premature birth due to foetal distress.[14] What is not clear is if this increased risk is as a result of underlying maternal illness, fever or placental insufficiency. There also seems to be a relationship between severity of the disease and premature birth, likely attributed to foetal stress as a result of maternal disease.[9] However, it is unclear what percentage of maternal body surface area needs to be denuded for the risk to become significant.

SJS/TEN is associated with mucosal surface necrosis and this is a major cause of long-term sequalae. Niemeijer et al reported genital involvement in 70% of patients which included; mucosal erosions, ulcerations and purulent blood-stained vaginal discharge. Mucosal lesions can persist for weeks to months and up to 28% of patients could suffer long term sequelae. These include vaginal adhesions and stenosis, vaginal and vulval adenosis, endometriosis and telangiectasia. Sequelae may result in painful intercourse, difficult conception and infertility[17]. In pregnancy this may interfere with normal delivery.[14]

The impact of HIV infection on maternal and foetal outcomes in pregnant women with SJS/TEN is largely unknown. It is well established that the HIV infected population has a higher predisposition to SJS/TEN.[2,8] In an attempt to answer some of these questions we conducted a review of clinical data and records of pregnant patients admitted to a single tertiary referral centre in South Africa with SJS/TEN and managed by the same group of dermatologists using the same protocols. We followed-up those who had delivered outside of the tertiary hospital by retrieving their records from secondary and primary level delivery facilities or contacting them physically and/or telephonically.

## Methods

### Study setting

The study population comprised of patients admitted with SJS/TEN to a tertiary referral hospital in Cape Town, South Africa. The hospital is one of two tertiary referral centres serving the population of Cape Town (approximately 3.7 million) including primary health care clinics, district and secondary hospitals[18]. The study was approved by the Faculty of Health Sciences Human Research Ethics Committee of the University of Cape Town (HREC Ref 124/2013) and conducted within the provisions of the World Medical Association Declaration of Helsinki.[19] The pregnant women were part of a prospective study on SJS/TEN effects on the foetus, new born and placenta (REC REF 81/2010). The placentas were collected post-delivery and sent for histology and the new-borns were examined for skin and mucosal anomalies. The women were also part of a prospective study of the long term mucosal sequelae of SJS/TEN (REC REF 82/2010). All patients gave consent for their data and information to be included prospectively in the RegiSCAR international registry for severe cutaneous adverse reactions to drugs (REC REF 424/2009). Additional information was obtained retrospectively from patient records and personal interviews where necessary. Signed informed consent was given by all women recruited into the prospective studies.

### Participants and data extraction

We reviewed clinical records and study data of all pregnant patients admitted with SJS, TEN and SJS/TEN overlap to the dermatology ward from 1^st^ January 2009 to 31^st^ December 2012. Patients were admitted to a general dermatology ward managed by experienced nursing staff and dermatologists in conjunction with obstetricians and gynaecologists where necessary. Patient management followed our standard protocol which is mainly supportive including fluid resuscitation, enteral nutritional support, daily baths with antiseptic solution, topical care of mucosa including eyes, mouth and genitalia and sterile non-adherent dressings. No physical debridement was performed and we avoided urinary catheters and intravenous lines unless intravenous antibiotics or urgent resuscitation was necessary. We avoided the use of antibiotics unless there was clinical or laboratory indication for their initiation. No prophylactic antibiotics, systemic steroids, intravenous immunoglobulin, cyclosporine or other specific therapeutic medications were administered to any of the patients. Anticoagulation was only administered to patients who were completely bed-bound for more than 7 consecutive days.

The following data parameters were extracted from the clinical records: age; HIV status; CD4 count; co-morbidities; gestation of pregnancy; medication used in the preceding 8 weeks; interval between the first symptoms of SJS/TEN and admission to our centre; classification as SJS, SJS/TEN overlap and TEN; vital signs; clinical management before and during admission to hospital; results of bacterial cultures; length of stay in hospital and the final outcome of both mother and child.

### Sampling and definitions

Where possible attempts to recover placentae at the time of delivery were made. These were collected in buffered formaline and sent for routine H&E staining. All placentas were reviewed by a single experienced pathologist as per our protocol.

### Data management and statistical analysis

Data was entered into a Microsoft Excel database. Continuous variables were described using medians and ranges or medians and interquartile ranges while categorical variables were summarized as proportions and percentages. No inferential statistics or hypothesis testing was undertaken as the small size of the sample did not allow for a meaningful analysis.

## Results

We identified twenty-two women (21 black African women and 1 woman of mixed ancestry) who developed SJS/TEN while pregnant and their baseline characteristics are summarised in **Table 1**. Their median age was 29 years (range 23–36) and all of the twenty-two were HIV-infected on antiretriviral therapy (ARV) with a median CD4 count of 316 (IQR 224,360). Apart from HIV, 3 participants had other pre-existing co-morbidities on admission namely; two cases of pre-eclampsia and a case of pneumonia. Eight of the twenty-two were primigravidas. SJS was diagnosed in 16/22 (73%) cases, TEN in 5/22 (23%) and SJS/TEN overlap in a single case. Seventeen of the twenty-two (77%) had genital erosions on admission and 2/17 (12%) of the cases had unspecified vaginal discharge. The median time between onset of symptoms and admission to hospital was four days (IQR 3,6). Nevirapine was the offending drug in 21/22 (95%) cases while efavirenz was implicated in a single case. Causality was decided clinically with reference to recently introduced drugs and experience and knowledge of the most likely offending agent. In all cases the ARVs given prophylactically to prevent HIV transmission to the child were the most recently introduced medications. Nevirapine regimes were used for all women except one where efavirenz was substituted for nevirapine. Onset of SJS/TEN symptoms was temporally related to the introduction of nevirapine (mean 16 days from nevirapine introduction) or to an increase in dose from 100mg to 200mg daily after 2 weeks of treatment (mean 4 days from increased nevirapine dose). In order to prevent the development of HIV resistance only nevirapine was stopped in suspected cases while the other ARVs were continued at full doses to cover the withdrawal tail of nevirapine excretion. In the case of efavirenz, all ARVs were stopped and on complete resolution of SJS/TEN the ARVs were restarted and efavirenz substituted with alluvia. Causality was thus determined by the temporal relationship to SJS/TEN and healing on withdrawal of the suspected offender despite continuation of all other medication.

**Table 1:** Baseline characteristics and description of pregnancy and outcomes of 22 HIV positive participants who developed SJS/TEN during pregnancy.

Patient	Age (years)	CD4 value	Diagnosis	Mucosal involvement	Past Obstetric History	Gestation at time of SJS/TEN (weeks+days)	Gestation at outcome (weeks+days)	Mode of delivery	Birth weight (grams)	Placental weight (grams)	Apgar 1min	Apgar 5min
1	37	360	SJS	Eye, oral, genital	G3P2	20 + 6	37 + 2	NVD	1950	450	9	10
2	24	887	SJS	Eye	G2P1	36	39 + 5	NVD	3415	480	8	9
3	34	110	SJS	Eye, oral	G2P1	37 +	41 + 2	C/S for high VL	3610	625	7	10
4	33	133	SJS	Eye,oral, geital	G2P0E1	29 + 3	39 + 3	NVD	3410	635	9	10
5	28	340	SJS	Eye, oral	G2P0M1	23 + 2	41	C/S	2565	350	9	10
6	32	1213	SJS	Eye,oral	G1P0	13	unknown	Unknown	Unknown	Unknown	Unknown	Unknown
7	36	26	SJS	Oral, genital	G3P2-1	24	31	NVD for IUD	580	170	0	0
8	33	236	SJS	Eye, oral, genital	G3P1M1	32 + 6	35 +	C/S for pre-eclampsia	2330	450	9	10
9	30	unknown	SJS	Eye,oral	G1P0	34	unknown	Unknown	Unknown	Unknown	Unknown	Unknown
10	26	162	TEN	Eye, oral, genital	G2P1	32	34 + 2	C/S for foetal distress	1760	Unknown	0	8
11	25	521	TEN	Eye, oral	G1P0	8	unknown	Unknown	Unknown	Unknown	Unknown	Unknown
12	28	622	SJS	Eye, oral, genital	G1P0	25	40 +	NVD	Unknown	Unknown	Unknown	Unknown
13	30	712	TEN	Eye, oral	G4P2M1	36 + 2	36 + 3	C/S for foetal distress	2230	430	9	10
14	24	203	SJS	Eye, oral, genital	G1P0	29 + 3	38 + 2	C/S for high VL	3040	425	7	10
15	23	224	SJS	Eye, oral	G2P1-1	14 +	39 +	C/S for foetal distress	2960	630	9	10
16	34	291	SJS	Eye, oral, genital	G3P2	23	39	NVD	3200	715	9	10
17	28	242	SJS/TEN	Eye, oral, genital	G1P0	27 +	unknown	Unknown	Unknown	Unknown	Unknown	Unknown
18	33	349	SJS	Eye, oral	G2P1	11	38 +	C/S for foetal distress	2520	450	8	9
19	27	348	SJS	Eye, oral, genital	G1P0	16	38	NVD	Unknown	Unknown	Unknown	Unknown
20	28	228	SJS	Eye	G2P1	15 + 3	39 +	NVD	3255	855	9	10
21	30	342	TEN	Eye, genital	G2P1	21	21 +	Miscarriage	330	100	0	0
22	26	345	TEN	Eye, oral genital	G1P0	28 + 6	39 + 2	C/S for foetal distress	2540	380	5	9

Key: **G**:gravidity, **P**:parity, **M**:miscarriage, **NVD**: normal vertex delivery, **C/S**: casaerian section, **VL**: viral

During hospitalization for SJS/TEN, deranged laboratory parameters (2xupper limit of normal) were reported in 7/22 (32%) and the most common was elevation of transaminases in four cases. One case each had elevated creatinine and alkaline phosphatase as well as a case of thrombocytopenia. Therapeutic systemic antibiotics were initiated in 13/22 cases, ten of these on clinical suspicion of bacterial systemic infection, which included temperature of >38°, tachycardia, offensive lochia, and three on confirmed positive bacterial cultures. The blood cultures grew *Klebsiella pneumoniae*,*methycillin*-susceptible *Staphylococcus aureus (S*.*aureus)* and methycillin-resistant *S*. *aureus* respectively. All the twenty two cases survived and were discharged home following recovery or delivery. No cases required admission to an intensive care unit (ICU). All cases were competently managed in an isolated side ward in the dermatology unit by a team of experienced nurses supported by a team of specialists. Management was based on the standardised unit protocol which dictates that all patients are monitored several times a day for sepsis, systemic involvement and metabolic imbalances. Three of the twenty-two (14%) developed post-partum sepsis (Table 2). The offending organism was known in only one patient, a case of methycillin-resistant *S*. *aureus*. Both cases of foetal death, one being a vertex delivery following an induction of labour and the other a miscarriage due to chorioamnionitis, were amongst the three that developed postpartum sepsis. The other case of post partum sepsis occurred after caesarean section delivery for foetal distress. Postpartum sepsis occurred a median of 17 days (range 12–29) after the initial presentation with SJS/TEN. Two of the three cases of post-partum sepsis occurred in women with SJS and the other in a woman with TEN. Only one of the cases had TEN at the time of delivery and sepsis, with the other 2 having recovered from their CADR before delivery. There were five reported cases of long-term sequelae associated with SJS/TEN in the study population, namely two cases of dyspareunia and two ophthalmological complications (Table 2). A fifth case was identified retrospectively as having a complication in her subsequent pregnancy in which she suffered 3^rd^ degree tears of the vagina.

**Table 2:** Baseline characteristics, offending agents and description of pregnancy with outcomes and complications of 22 HIV positive participants who developed SJS/TEN in pregnancy.

Pt	Age (yrs)	CD4	VL	CADR	Drug	Days to CADR	Days to admission	Co-morbidity	GA (weeks)	U/S	Antibiotics	Antibiotic indication	Septic	Pregnancy outcome	Foetal complications	Antenatal Complications	Maternal Complications	Child’s 6 week HIV PCR
**1**	37	360	LDL	SJS	NVP	15	4	None	20+6	N	Cef	clinical	no	Alive, NVD, IOL for IUGR	IUGR	IUGR Chronic diarrhoea	Eye problems	Neg
**2**	24	887	<40	SJS	EFV	17	9	None	36	N	0	0	yes	Alive, NVD	none	None	None	Neg
**3**	34	110	1260	SJS	NVP	15	10	None	37+1	N	Cotrimox; Metroni	clinical	yes	Alive, C/S for high VL	none	None	None	Neg
**4**	33	133	<40	SJS	NVP	22	4	None	29+3	N	0	0	yes	Alive, NVD	none	None	None	neg
**5**	28	340	<40	SJS	NVP	19	3	None	23+2	N	0	0	no	Alive, C/S	none	None	None	UNK
**6**	32	1213	LDL	SJS	NVP	15	2	Pneumonia	13	ND	Amoxyl	clinical	no	Alive, UNK	UNK	UNK	UNK	UNK
**7**	36	26	UNK	SJS	NVP	8	6	None	24	N	0	0	yes	Dead, NVD, IOL for IUD	IUD	Herpes Simplex	Postpartum Sepsis	N/A
**8**	33	236	LDL	SJS	NVP	10	4	pre-eclampsia	32+6	N	Clinda	clinical	no	Alive, C/S for preeclampsia	none	Pre-eclampsia	UNK	Neg
**9**	30	UNK	UNK	SJS	NVP	26	1	None	34	ND	Amoxyl, Augmentin	LRTI	no	UNK	UNK	UNK	UNK	UNK
**10**	26	162	UNK	TEN	NVP	14	7	None	32	N	Ampi, Metroni, Erta, Amik	clinical; LRTI; URTI	no	Alive, C/S for foetal distress	Birth asphyxia LBW	RLL pneumonia	UNK	Neg
**11**	25	521	UNK	TEN	NVP	13	3	None	8	ND	Cef, Erythro, Augmentin,Amoxyl	clinical	no	UNK	UNK	UNK	UNK	UNK
**12**	28	622	UNK	SJS	NVP	22	6	None	25	ND	0	0	no	Alive, NVD	none	UNK	Dyspareunia	Neg
**13**	30	712	LDL	TEN	NVP	UNK	3	None	Post-partum	N	Clinda	clinical	no	Alive, C/S for foetal distress	congenital pneumonia	none	UNK	Neg
**14**	24	203	<40	SJS	NVP	15	4	None	29+3	N	Ampi, Cef	clinical	no	Alive C/S for high VL	none	Liver impairment	UNK	Neg
**15**	23	224	LDL	SJS	NVP	18	7	None	14+1	N	0	0	no	Alive C/S for foetal distress	Failed IOL	none	Postpartum sepsis	UNK
**16**	34	291	UNK	SJS	NVP	24	3	None	23	ND	Clinda	clinical	no	Alive, NVD	none	None	None	Neg
**17**	28	242	LDL	SJS/TEN	NVP	19	1	None	27+1	N	0	0	no	UNK	UNK	UNK	UNK	UNK
**18**	33	349	1.54	SJS	NVP	14	10	None	11	N	0	0	no	Alive C/S for foetal distress	respiratory distress	Pre-eclampsia	Eye problems	UNK
**19**	27	348	UNK	SJS	NVP	12	2	None	16	ND	0	0	no	Alive, NVD	None	UNK	None	Neg
**20**	28	228	<40	SJS	NVP	18	4	None	15+3	N	0	0	no	Alive, NVD	None	None	None	Neg
**21**	30	342	UNK	TEN	NVP	1	5	None	21	IUD	Vanco	3	no	miscarriage, medical TOP	miscarriage	Chorio-amnionitis	Postpartum sepsis	N/A

Key: Amik: amikacin, Ampi: ampicillin, CADR: cutaneous adverse drug reaction, Cef: ceftriaxone, Clinda: clindamycin, Cotrimox: cotrimoxazole, C/S: caesarean section, EFV: efavirenz, Erta: ertapenem, Erythro: erythromycin, GA: gestational age, IOL: induction of labour, IUD: intra-uterine death, IUGR: intra-uterine growth restriction, LDL: lower than detectable limit, LRTI: lower respiratory tract infection, Metroni: metronidazole, N: normal, ND: not done, Neg: negative, NVP: Nevirapine, NVD: normal vertex delivery, RLL: right lower lobe, UNK: unknown, UTI: urinary tract infection, Vanco: vancomycin

Outcomes of the pregnancy were known in 18/22 women. Seven of the eighteen pregnancies (39%) had a normal vertex delivery while 9/18 (50%) patients were delivered by caesarean section. Two of the eighteen pregnancies (11%) resulted in intrauterine deaths (**Tables 1 and 2**). Six of the nine caesarean sections were emergencies, the indications being foetal distress in three cases, increased maternal viral load in two cases and a case of worsening pre-eclampsia. The two foetal deaths were a second trimester miscarriage at 148 days (21+1 weeks) and the other an intrauterine foetal death at 217days (31 weeks). Both had non-viable birthweights of 330g and 580g respectively. The placenta to birthweight ratios were both 0.3, well within normal limits. The median gestation at delivery of the live births was 274 days (39weeks) (IQR: 265, 277) and the median birth weight was 2763g (IQR: 2378, 3241). Placental weights were available for 13/18 (72%) of the cases and the median placenta to foetal weight ratio was 0.18 (range 0.14–0.26) (**Table 1)**. Apgar scores were available for 14/18 (78%) live births. The one minute score was 9 in 8/14 cases (57%), 8 in 2/14 (14%), 7 in 2/14 (14%) and 5 and 0 in 1/14 case each. At five minutes the Apgar scores were 10 for 10/14 (71%), 9 for 3/14 and 8 for single case. There were complications in 5/16 (31%) of the live deliveries. Three of the cases involved the respiratory system, namely birth asphyxia and congenital pneumonia. The other two cases were low birth weight babies weighing 1760g and 1950g respectively. Two of the five live birth complications occurred in mothers who had experienced TEN (2 and 15 days after the CADR). Three were associated with mothers who had had SJS. These complications however occurred more than 6weeks after the CADR (6weeks, 4months and 6months). Eight placentae and one foetus were sent for macroscopic and histological evaluation. No abnormalities of the placental surface, membranes, vasculature and chorionic villi were detected. The foetus showed no macroscopic or microscopic features of SJS/TEN.

All new-borns received prophylactic ARVs (nevirapine) until HIV status confirmation at 6 weeks post-delivery without incident. On follow-up, results of 6week HIV PCRs were only available for twelve of the twenty children. None of them were HIV-infected. Maternal CD4 counts at the time of pregnancy were available for ten of these twelve women, with the median being 262 cells/mm^3^ (IQR: 210,391). Viral loads were available for eight of the mothers of the children who were known to have been tested (Table 2). Seven of the mothers were adequately supressed, 4 <40 copies/ml in four and lower than the detectable limit in three. Two women were delivered by caesarean section for high maternal viral load, one with a viral load of 1260 copies/ml and the other with a presumed high viral load due to late start of ARV therapy but no viral load was documented prior to caesarean section.

## Discussion

In this series of 22 consecutive women who developed SJS/TEN during their pregnancy, to our knowledge the largest published single centre experience, our main findings were: a) SJS/TEN-associated mortality is not increased in HIV-infected pregnant women b) maternal SJS/TEN does not manifest in the foetus c) SJS/TEN in pregnancy is not associated with intra-uterine HIV transmission.

It is well established that the incidence of SJS/TEN is increased in HIV-infected persons, however it is not clear if SJS/TEN-associated mortality is higher in this population. A literature review by Struck and colleagues suggested a high survival rate for the mother (24/28 survived, 1/28 died, 3/28 unknown outcome). Foetal survival was not as high (22/28 survived, 5/28 died, 1/28 unknown outcome).[14] Among our participants, maternal mortality was zero and none of the women required ICU admission. The zero maternal mortality was due to dedicated intensive nursing and supportive care without use of any immunomodulatory therapies. Foetal mortality was 2/18 (11%) documented pregnancy outcomes, marginally better than the 5/27 (19%) reported in the review. Our maternal and foetal mortality figures confirm and strengthen those reported in Struck and colleagues review as we report consecutive cases treated at a single centre following a standard treatment protocol. Due to the rarity of the disease and the small study population, it is difficult to quantify mortality associated with SJS/TEN in pregnant predominantly HIV-infected women. In our experience mortality was zero, with further possible explanations being that pregnant women have fewer co-morbidities and as a result are on less concomitant medications and they are younger. All were below the age of 40 years in our series, an age that is considered a low risk factor for mortality according to SCORTEN.[13] This is supported by comparison with larger reported series which showed comparatively lower mortality in children and younger cohorts. [5,8,12,20–26]

There have been previous reports of SJS/TEN manifesting in both the mother and the foetus when the disease occurs during pregnancy.[15] In our series of 22 consecutive patients none of the foetuses, both intrauterine deaths and live births, showed clinical features of SJS/TEN. This, taken together with existing English literature of only 1 published cases of mother and child being affected, suggests that foetal manifestation of SJS/TEN are rare during pregnancy.[15] Placenta-to-birth weight ratio (PWR) is variable and depends on numerous factors including race, gestational age, anaemia, maternal body mass, age, smoking and presence of pregnancy-related pathology.[27,28] PWR has been shown to be predictive of obstetrical outcomes as well as perinatal morbidity and mortality. PWR can be thought of as a balance between foetal and placental growth.[29] In this cohort of pregnant women the median PWR was within the normal ranges reported in the literature (mean 0.2 SD = 0.044, minimum 0.023, maximum 1.17).[29] A total of 8/22 placentas were examined histologically and no abnormalities were found. However, these histology findings should be interpreted with caution due to a significant possibility of a sampling error in assessing histological sections of such a large tissue mass. Despite this, it seems that SJS/TEN, although a severe maternal disease, does not have a significant impact on PWR and the physical structure of the placenta. The possible explanations include the relatively short duration of the SJS/TEN; onset of the disease late in pregnancy when the disease is less likely to have a sustained impact on PWR; and confinement of SJS/TEN to the mother, without direct involvement of the placenta and foetus. Larger studies, stratifying the study population based on the above-mentioned parameters, are needed to clarify the impact of SJS/TEN on PWR.

A recent report of an uneventful normal vaginal delivery after a well monitored pregnancy following recovery from SJS highlights the importance of genital care in SJS/TEN.[30] In our cohort, none of the women developed clinically detectable labial, vulvar or vaginal adhesions on pelvic examination. This is likely to be a result of our routine practice of four times a day Sitz baths with separation and lubrication of apposed eroded mucosa surfaces in patients with genital lesions. Vaginal stenosis and labial synechiae result from adhesions of apposing denuded mucosal surfaces which results in fibrosis. Before this practice we had encountered a few cases of vaginal stenosis and fibrosis of the labia. No formal examination of the genito-urinary tract were performed on these patients, thus it is still possible we missed these well established sequelae of SJS/TEN, particularly in the cases delivered by caesarian section for foetal distress or other indications.[30–32]

Nevirapine was the most common offending drug in 21 of the 22 cases. The use of nevirapine in prevention of mother to child transmission of HIV (PMTCT) regimens during the study period was the major contributor to the higher incidence of nevirapine-associated SJS/TEN in this population. We expect a significant drop in the incidence of SJS/TEN with elimination of nevirapine from the ARV protocols that have recently been introduced in South Africa.

As a result of the small number of TEN cases and loss to follow up of these cases we could not establish that TEN, the more severe form of the disease, is associated with poorer foetal outcomes. Larger studies involving multiple centres are required to determine if severity of SJS/TEN impacts maternal and/or foetal outcomes in pregnant women.

There are several limitations to our study. Some of the patients delivered in secondary hospitals hence the loss to follow up and missing data. Another limitation of the study as with others on this subject is the small number of subjects in the study population precluding a meaningful statistical analysis. However, all the patients were consecutive and managed at a single center during the acute stage of SJS/TEN using similar protocols allowing for a cohesive assessment of the data.

## Summary

We describe clinical characteristics and outcomes of SJS/TEN in a series of consecutive HIV-infected pregnant women. We found that maternal mortality was not increased, probably lower, in this population compared to the general population. SJS/TEN did not affect the placenta and foetus directly. SJS/TEN did not increase the risk of HIV transmission to the foetus in mothers on ARV prophylaxis.
